# Anti-SARS-CoV-2-specific antibodies in human breast milk following SARS-CoV-2 infection during pregnancy: a prospective cohort study

**DOI:** 10.1186/s13006-023-00605-w

**Published:** 2024-01-18

**Authors:** Irene Fernández-Buhigas, Nieves Rayo, Julia Cuesta Silos, Berta Serrano, Olga Ocón-Hernández, Bo Wah Leung, Juan Luis Delgado, David Sánchez-Nieves Fernández, Silvia Valle, Laura De Miguel, Aroa Silgado, Ramón Perez Tanoira, Valeria Rolle, Belén Santacruz, Maria M. Gil, Liona C. Poon

**Affiliations:** 1 Obstetrics and Gynecology Department, Hospital Universitario de Torrejón, Torrejón de Ardoz, Madrid, Spain; 2 Vicerrectorado de Investigación, Facultad de Medicina, Universidad Francisco de Vitoria, Carretera Pozuelo a Majadahonda, Km 1.800, Pozuelo de Alarcón, Madrid, 28223 Spain; 3 Synlab Diagnósticos Globales S.A., Esplugues de Llobregat, Catalonia Spain; 4 Department of Obstetrics, Hospital Universitari Vall d’Hebron, Universitat Autònoma de Barcelona, Barcelona, Catalonia Spain; 5 Obstetrics and Gynecology Department, Hospital Universitario Clínico San Cecilio, Granada, Spain; 6 Instituto de Investigación Biosanitaria Ibs, Granada, Spain; 7 Department of Obstetrics and Gynaecology, The Chinese University of Hong Kong, Hong Kong SAR, China; 8 Obstetrics and Gynecology Department, Hospital Clínico Universitario Virgen de La Arrixaca, El Palmar, Murcia, Spain; 9 Obstetrics and Gynecology Department, Hospital Universitário Príncipe de Asturias, Alcalá de Henares, Madrid, Spain; 10 Universidad de Alcalá de Henares, School of Medicine, Alcalá de Henares, Madrid, Spain; 11 Department of Microbiology, Hospital Universitari Vall d’Hebron, Universitat Autònoma de Barcelona, Barcelona, Catalonia Spain; 12 Department of Microbiology, Hospital Universitário Príncipe de Asturias, Alcalá de Henares, Madrid, Spain; 13 Biostatistics and Epidemiology Platform at Instituto de Investigación Sanitaria del Principado de Asturias, Oviedo, Asturias Spain

**Keywords:** Human Breast Milk, Colostrum, SARS-CoV-2, COVID-19, Anti-SARS-CoV-2 specific antibodies

## Abstract

**Background:**

While the presence of SARS-CoV-2 in human breast milk is contentious, anti-SARS-CoV-2 antibodies have been consistently detected in human breast milk. However, it is uncertain when and how long the antibodies are present.

**Methods:**

This was a prospective cohort study including all consecutive pregnant women with confirmed SARS-CoV-2 infection during pregnancy, recruited at six maternity units in Spain and Hong Kong from March 2020 to March 2021. Colostrum (day of birth until day 4 postpartum) and mature milk (day 7 postpartum until 6 weeks postpartum) were prospectively collected, and paired maternal blood samples were also collected. Colostrum samples were tested with rRT-PCR-SARS-CoV-2, and skimmed acellular milk and maternal sera were tested against SARS-CoV-2 specific immunoglobulin M, A, and G reactive to receptor binding domain of SARS-CoV-2 spike protein 1 to determine the presence of immunoglobulins. Then, we examined how each immunoglobulin type in the colostrum was related to the time of infection by logistic regression analysis, the concordance between these immunoglobulins in the colostrum, maternal serum, and mature milk by Cohen's kappa statistic, and the relationship between immunoglobulin levels in mature milk and colostrum with McNemar.

**Results:**

One hundred eighty-seven pregnant women with confirmed SARS-CoV-2 infection during pregnancy or childbirth were recruited and donated the milk and blood samples. No SARS-CoV-2 was found in the human breast milk. Immunoglobulin A, G, and M were present in 129/162 (79·6%), 5/163 (3·1%), and 15/76 (19·7%) colostrum samples and in 17/62 (27·42%), 2/62 (3·23%) and 2/62 (3·23%) mature milk samples, respectively. Immunoglobulin A was the predominant immunoglobulin found in breast milk, and its levels were significantly higher in the colostrum than in the mature milk (*p*-value < 0.001). We did not find that the presence of immunoglobulins in the colostrum was associated with their presence in maternal, the severity of the disease, or the time when the infection had occurred.

**Conclusions:**

Since anti-SARS-CoV-2 antibodies are found in the colostrum irrespective of the time of infection during pregnancy, but the virus itself is not detected in human breast milk, our study found no indications to withhold breastfeeding, taking contact precautions when there is active disease.

**Supplementary Information:**

The online version contains supplementary material available at 10.1186/s13006-023-00605-w.

## Background

On 11 March 2020, the COVID-19 pandemic was declared by the World Health Organization (WHO) [1]. Since then, extensive efforts have focused on evaluating the effects of the new coronavirus on pregnancy. At the very beginning of the pandemic, newborns were separated from their mothers with confirmed SARS-CoV-2 infection to protect them against the virus. Breastfeeding was avoided because it was unknown if the virus could be transmitted via human breast milk. To date, some studies have reported the presence of SARS-CoV-2 in human breast milk [2–7] while others have not [8–14], but the sample size of these studies is small.

Currently, most healthcare systems and international organizations such as the Centers for Disease Control and Prevention (CDC) recommend breastfeeding for all mothers with active or past infection of SARS-CoV-2, as there appear to be more benefits of breastfeeding than the potential risk of transmission through human breast milk. One of the most important reasons to recommend breastfeeding is the possible passive immunization in newborns against SARS-CoV-2 [15]. In particular, IgA is important because it coats and seals the neonate's respiratory and intestinal tracts to prevent microorganisms from entering the body and ​bloodstream, constituting the first defense against the virus [16, 17]. Several studies have reported the presence of anti-SARS-CoV-2 antibodies [18–25] in human breast milk. Pace et al. have demonstrated that the specific IgG, IgM, and IgA anti-SARS-CoV-2 antibodies in human breast milk can effectively neutralize SARS-CoV-2 infectivity [11]. However, it is uncertain when the antibodies become present and how long they last in human breast milk.

The aims of this study were first, to determine the presence of anti-SARS-CoV-2 virus and antibodies in colostrum and mature human breast milk in women who had SARS-CoV-2 infection during pregnancy or at the time of childbirth; second, to investigate the association between the anti-SARS-CoV-2 antibodies in human milk with the levels of anti-SARS-CoV-2 antibodies in maternal blood, the severity of SARS-CoV-2 infection and the time interval from active illness; and third, to evaluate how each immunoglobulin type evolved from the colostrum to the mature milk.

## Methods

### Study population

This was a prospective cohort study aiming to include all consecutive pregnant women with laboratory-confirmed SARS-CoV-2 infection by deep throat saliva (DTS) or nasopharyngeal swab (NPS) real-time reverse-transcriptase-polymerase-chain-reaction (rRT-PCR) test or by rapid antigen-detection tests (Panbio™ COVID-19 Ag Rapid Test Device) [26], during pregnancy, labor or immediately after childbirth, who were able to provide consent to participate in the study, from six maternity units, five in Spain (Hospital Universitario de Torrejón and Hospital Universitario Príncipe de Asturias in Madrid, Hospital Universitario Vall d'Hebrón in Barcelona, Hospital Clínico Universitario San Cecilio in Granada and Hospital Clínico Universitario Virgen de la Arrixaca in Murcia) and one in Hong Kong SAR, China (The Chinese University of Hong Kong COVID-19 collaborative network), from March 2020 to March 2021. Eligibility criteria were: confirmed SARS-CoV2 infection, over 18 years old, and fluent in the investigator's language. Additionally, for suspected cases of COVID-19 where rRT-PCR was negative, if the symptoms had started within seven days of testing, the rRT-PCR was repeated 24 h after the first test. If the symptoms had started beyond seven days of testing, a serology test (ELISA) was performed [27] and women with positive results by either test were also offered participation.

Breast milk samples were collected from the six maternity units. All participants were unvaccinated against SARS-CoV-2, and it was their first SARS-CoV-2 infection.

The Strengthening the Reporting of Observational Studies in Epidemiology (STROBE) Statement was used for reporting the results (Additional file. Table 1s).

**Table 1:** Maternal, pregnancy, and disease characteristics according to time of infection and disease activity

	**Infection at the First trimester** (***N*** = 38)	**Infection at the Second trimester** (***N*** = 65)	**Infection at the Third trimester** (***N*** = 57)	**No active disease** (***N*** = 158)	Active disease at birth ***(N*** = 31)	**Overall** (*N* = 189)
Maternal age (years)	33.0 [30.3, 37.0]	34.0 [30.0, 37.0]	31.0 [28.0, 36.0]	33.0 [29.0, 37.0]	33.0 [28.5, 37.0]	33.0 [29.0, 37.0]
Height (cm)	165 [159, 170]	162 [159, 168]	161 [156, 168]	163 [158, 169]	161 [156, 166]	163 [158, 168]
Weight (kg)	65.0 [56.3, 74.8]	69.0 [58.0, 76.0]	62.5 [54.0, 70.3]	65.0 [56.5, 74.0]	60.0 [54.4, 74.0]	64.5 [56.0, 74.0]
Missing	0 (0%)	0 (0%)	1 (1.8%)	1 (0.6%)	0 (0%)	1 (0.5%)
Body mass index (kg/m^2^)	24.2 [21.5, 27.5]	24.9 [21.5, 28.7]	23.4 [21.7, 25.8]	24.1 [21.5, 27.6]	24.7 [21.0, 28.7]	24.2 [21.5, 28.0]
COVID-19 Diagnosis with						
Antigens	9 (23.7%)	13 (20.0%)	7 (12.3%)	29 (18.4%)	3 (9.68%)	32 (16.9%)
RRT-PCR	20 (52.6%)	38 (58.5%)	38 (66.7%)	94 (59.5%)	26 (83.9%)	120 (63.5%)
Serology	9 (23.7%)	14 (21.5%)	12 (21.1%)	35 (22.2%)	2 (6.45%)	37 (19.6%)
Gestational age at diagnosis of COVID-19 (days)	65.5 [50.5, 82.8]	162 [132, 184]	240 [226, 254]	176 [105, 226]	268 [265, 280]	195 [115, 248]
COVID-19 symptoms						
Asymptomatic	6 (15.8%)	8 (12.3%)	17 (29.8%)	30 (19.0%)	17 (54.8%)	47 (24.9%)
Mild	31 (81.6%)	50 (76.9%)	33 (57.9%)	113 (71.5%)	12 (38.7%)	125 (66.1%)
Pneumonia	1 (2.63%)	7 (10.8%)	7 (12.3%)	15 (9.49%)	2 (6.45%)	17 (8.99%)
Gestational age at birth (days)	279 [270, 284]	277 [272, 283]	278 [274, 283]	278 [273, 284]	275 [268, 286]	278 [272, 284]

Results are presented as median (interquartile range) or as n (%) as appropriate

Participants had one sample of colostrum (between the day of birth and day 4 postpartum) collected and stored at -80ºC. Maternal blood for serological analysis was also collected simultaneously; serum was separated and stored at -80ºC. One sample of fore mature milk (from day 7 to 6 weeks postpartum) was also collected and stored whenever possible.

Clinical data, including maternal age, body mass index (BMI) at the beginning of pregnancy, gestational age at the time of SARS-CoV-2 infection, and disease severity, were recorded for every participant, pseudo-anonymized, and entered into a secured common database. The COVID-19 severity was classified as asymptomatic, mild (when no hospitalization was required), and severe (when the diagnosis of pneumonia was established and hospitalization was needed) [28]. Gestational age was determined by first trimester sonographic assessment of fetal crown-rump length [29] or conception date in vitro fertilization pregnancy.

### Biological sample collection and analysis

Breast milk (from 0.1 to 1.0 mL) was collected by manual expression with strict contact precautions to avoid contamination (facial mask and hand cleaning). Blood samples were collected in serum sep clot activator 8 mL tubes, centrifuged for five minutes at 3500 g, and then serum was collected. Both serum and breast milk samples were divided into 0.5 mL aliquots (when possible) in separate Eppendorf tubes, labeled with a unique patient identifier, and stored at -80ºC until subsequent analysis. Stored samples from Barcelona were analyzed locally at the end of the recruitment period. Samples from all other sites were sent without any further processing overnight on dry ice to Synlab Diagnósticos Globales Laboratory in Madrid every month from Spanish sites and in a single batch after rt-RT-PCR testing was performed locally at the end of the recruitment period from Hong Kong.

Breast milk samples were thawed at the laboratory and centrifuged at 800 g for 15 min. Fat was removed, and the supernatant was transferred to a new tube. Centrifugation was repeated twice to ensure the removal of all cells and fat [22]. Skimmed acellular milk was then tested against SARS-CoV-2 specific immunoglobulin M (IgM), immunoglobulin A (IgA), and immunoglobulin G (IgG) reactive to the receptor binding domain (RBD) of the SARS-CoV-2 spike protein 1 (protS1) [22]. As previously reported, serum samples were thawed and tested against SARS-CoV-2 specific antibodies. All equipment and reagents used for analyses are CE (Conformité Européenne) marked (Additional file. Table 2s).

**Table 2:** Number of colostrum, mature milk, and maternal blood samples and proportions with anti-SARS-CoV2 virus or antibodies detected by semiquantitative analysis (reported as a ratio)

Analysis	Colostrum	Mature milk	Maternal blood at birth
**IgA**			
Indeterminate (0·8 to 1·1)	4 (2.14%)	0 (0%)	15 (12.5%)
Negative (< 0·8)	29 (15.5%)	51 (75.0%)	42 (35.0%)
Positive (> 1·1)	129 (69.0%)	15 (22.1%)	62 (51.7%)
Insufficient sample	25 (13.4%)	2 (2.94%)	1 (0.833%)
Missing	2 (1.1%)	121 (64.0%)	69 (36.5%)
Total simples analysed	187	68	120
**IgG**			
Indeterminate (0·8 to 1·1)	3 (1.60%)	0 (0%)	9 (6.47%)
Negative (< 0·8)	155 (82.9%)	63 (92.6%)	61 (43.9%)
Positive (> 1·1)	5 (2.67%)	3 (4.41%)	68 (48.9%)
Insufficient sample	24 (12.8%)	2 (2.94%)	1 (0.719%)
Missing	2 (1.1%)	121 (64.0%)	50 (26.5%)
Total simples analysed	187	68	139
**IgM**			
Indeterminate (0·9 to 1·1)	0 (0%)	0 (0%)	4 (4.26%)
Negative (< 0·9)	61 (60.4%)	52 (96.3%)	59 (62.8%)
Positive (> 1·1)	15 (14.9%)	0 (0%)	30 (31.9%)
Insufficient sample	25 (24.8%)	2 (3.70%)	1 (1.06%)
Missing	88 (46.6%)	135 (71.4%)	95 (50.3%)
Total simples analysed	101	54	94
**rRT-PCR**			
Negative	73 (90.1%)		
Positive	0 (0%)		
Inhibited	3 (3.70%)		
Insufficient sample	5 (6.17%)		
Missing	108 (57.1%)		
Total simples analysed	81		

A semiquantitative method was used. For this analysis, the ratio between the extinction of the control or patient sample and the extinction of the calibrator is calculated according to the following formula: Extinction of the control or patient sample / Extinction of calibrator

Extinction refers to the Optical Density or Absorbance at 450nm Wavelength

The extinction of the calibrator defines the upper limit of the reference range of non-infected persons (cut-off or threshold) recommended by the manufacturer. Values above the indicated cut-off are considered positive and those below negative

*IgA* Specific anti-SARS-CoV2 immunoglobulin A, *IgG* Specific anti-SARS-CoV2 immunoglobulin G, *IgM* Specific anti-SARS-CoV2 immunoglobulin M), rRT-PCR Real-time reverse-transcriptase-polymerase-chain-reaction

#### Immuno-analyses

- Determination of IgA and IgG antibodies was performed by the ELISA method (Enzyme-Linked Immunosorbent Assay), providing semiquantitative serology results against the S1 domain of the spike protein of SARS-CoV-2 in serum samples (Anti-SARS-CoV-2 ELISA IgG and Anti-SARS-CoV-2 ELISA IgA, Euroimmunn Medizinische Labordiagnostika AG, Lubeck, Germany) [30, 31]. Semiquantitative results were calculated as extinction of the control patient sample/extinction of calibrator (further details on this type of analysis are provided in Table 2). IgA and IgG were considered positive, indeterminate, and negative when results were > 1.1, 0.8 to 1.1 and < 0.8, respectively, as recommended by the manufacturer.
- IgM determination was performed with chemiluminescence microparticle immunoassays, using spike protein-specific (Abbott test, SARS-CoV-2 IgM Abbott, Abbott Ireland Diagnostics Division Finisklin, Ireland) [32], providing semiquantitative (extinction of the control patient sample/extinction of calibrator). IgM was considered positive, indeterminate, and negative when results were > 1.1, 0.9 to 1.1 and < 0.9, respectively, as recommended by the manufacturer.

#### rRT-PCR-SARS-CoV-2 testing

Whenever available, a second colostrum aliquot was tested for SARS-CoV-2 by rRT-PCR to assess the presence of the virus in the sample. In the Spanish samples, viral RNA was extracted with Chemagic Viral DNA/RNA Kit using the Chemagic 360 with integrated dispense, which includes lyophilized Poly(A) RNA, lyophilized Proteinase K, and a lysis/binding buffer, and were analyzed with Euroinmune Kit (ORF1ab an N targets) and TaqMan™ 2019-nCov Assay kitv2 Thermofisher (s,ORF1ab and N targets). In the Hong Kong samples, viral RNA was extracted using RNeasy® Mini Kit (QIAGEN), and the detection of SARS-CoV-2 RNA was performed with the FDA-authorized CDC 2019-Novel Coronavirus (2019 nCoV) Real-Time RT-PCR Diagnostic Panel (EUA 200001). The N gene (N1 and N2) was assayed, with the human RNase P (RP) as an endogenous reference control. In all cases, samples containing organic or inorganic contaminants interfering with the PCR amplification process were considered inhibited (these samples contained organic or inorganic contaminants that interfered with the PCR amplification process).

For this study, we included all women with available colostrum; additional samples or analyses were not mandatory for inclusion. Given the limited volume of colostrum and serum collected, not all tests could be carried out in all cases. For some laboratory analyses that failed at the first attempt, repeat testing was not possible. Besides, many women did not return to the clinic after birth due to the lockdown. Therefore, we could not collect mature milk in these cases.

### Statistical analysis

Descriptive data were expressed as median and interquartile range (IQR) and in proportions (absolute and relative frequencies). Cohen's Kappa was used to assess the colostrum and serum concordance. The kappa statistic was calculated without weighting; very good levels of agreement were considered when it is > 0.80, good 0.80–0.60, moderate 0.60–0.40, poor 0.40–0.20 and very poor < 0.20 [33]. Univariable logistic regression analysis was performed to assess if the presence of immunoglobulins in colostrum was associated with the presence of immunoglobulins in maternal serum, the severity of maternal symptoms, or the time passed from infection. Odds ratio (OR) and 95% confidence interval (CI) were calculated [34]. Lastly, the McNemar test was used to evaluate how each immunoglobulin type evolved in all paired colostrum-mature milk samples; this test reports *p* values based on the chi squared distribution with 1 degree of freedom. The level of significance was set at 0.05.

The statistical software R version 4.1.2 (Vienna, Austria) was used for all data analyses [35].

## Results

**Fig. 1:**
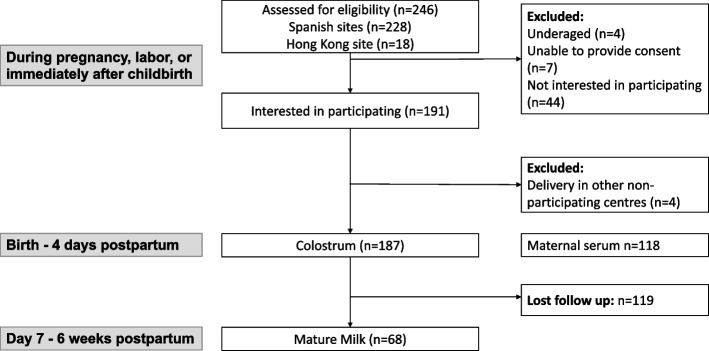
Sample flow chart. STROBE layout

**Fig. 2:**
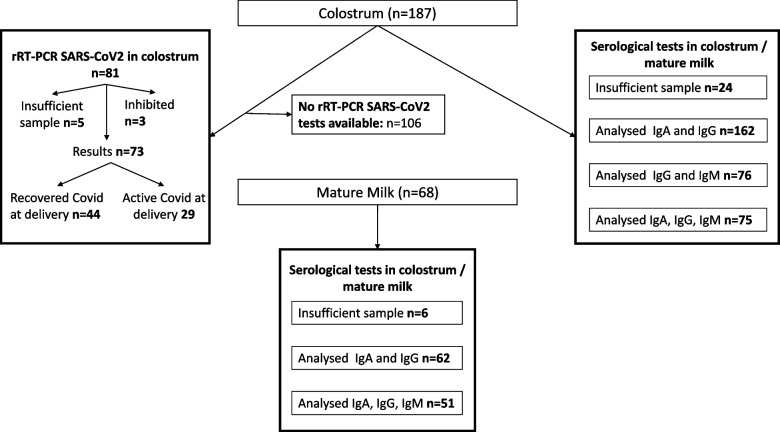
Sample analysis Flow chart

A total of 246 pregnant women with confirmed SARS-CoV2 infection during pregnancy or childbirth were eligible and were approached with information about the study. After exclusions, 191 women agreed to participate (4 were underaged, 7 were unable to provide consent, and 44 were not interested in participating). Among those, 187 had colostrum available for analysis (Figs. 1, 2). Of these, 38 (20.3%), 65 (34.8%), and 84 (44.9%) women acquired the infection in the first (< 14 weeks), second (14–28^+6^), and third trimester (> 28^+6^) of pregnancy, respectively. Among the cases with third-trimester infection, 29 (34.5%) had active SARS-CoV-2 infection at childbirth (rRT-PCR-SARS-CoV-2 positive at birth). Pregnancy and disease characteristics are shown in Table 1.

The colostrum and blood samples were collected between the day of birth and day 4 postpartum (median = 1; IQR 0 to 1). Mature milk samples were collected after day 7 postpartum (median = 39 days, IQR 25 to 44). Sample availability and serological status are displayed in Table 2.

Out of the 187 samples collected, only 162 yielded results for IgA, 163 for IgG, and 76 for IgM due to technical issues such as limited volume and assay failures (Table 3). IgA, IgG, and IgM were present in 129/162 (79.6%), 5/163 (3.1%), and 15/76 (19.7%) colostrum samples, respectively. All immunoglobulin-positive colostrum samples tested positive for IgA, except for one sample that only tested positive for IgG (IgA and IgM negative). Another tested positive for IgM and IgG, but there was insufficient sample for the detection of IgA. None of the samples had all 3 immunoglobulins detected.

**Table 3:** Colostrum serology results according to type of immunoglobulin

IgA (187)	IgG (187)	IgM (101)	Number of samples
Positive	Positive	Negative	2
		No result	2
	Negative	Positive	14
		Negative	39
		No result	70
	Indeterminate	No result	2
Negative	Positive	Negative	1
	Negative	Negative	17
		No result	10
	Indeterminate	No result	1
Indeterminate	Negative	Negative	2
		No result	2
No result	Negative	Positive	1

*IgA* Specific anti-SARS-CoV2 immunoglobulin A, *IgG* Specific anti-SARS-CoV2 immunoglobulin G, IgM Specific anti-SARS-CoV2 immunoglobulin M). No result: when no test result was obtained for that immunoglobulin analysis

Seventy-six colostrum samples were tested for rRT-PCR-SARS-CoV-2, including 29 with active disease at birth. 73 tested negative, and 3 were inhibited (these samples contained organic or inorganic contaminants that interfered with the PCR amplification process).

### Association between colostrum and serum

One hundred eighteen women had at least one serology result. Association between the colostrum and serum measured with Cohen's Kappa was 0.09 (CI 95% -0.11 to 0.30) for IgA; 0.06 (CI 95% -0.01 to 0.12) for IgG, and 0.29 (CI 95% 0.03 to 0.54) for IgM (Table 4).

**Table 4:** IgM, IgA and IgG results for colostrum and delivery serum samples

	**Antibody results at delivery**			
		**Negative**	**Positive**	**Total**
**Antibody results in colostrum**	**IgM**			
	*Negative*	41	11	52
	*Positive*	6	7	13
	*Total*	47	18	65
	**IgA**			
	*Negative*	8	9	17
	*Positive*	23	43	66
	*Total*	31	52	83
	**IgG**			
	*Negative*	57	50	107
	*Positive*	0	3	3
	*Sum*	57	53	110

### Factors related to colostrum positivity

There were no statistically significant differences between the immunoglobulin status in colostrum and the severity of the symptoms nor the time interval from the disease, either as a continuous variable or considering only active disease at birth vs. no active disease at birth (Table 5).

**Table 5:** Factors related to colostrum positivity. Results from three univariable logistic regression models to identify significant predictors of immunoglobulin positivity in colostrum among a) symptoms, b) interval from disease to sample, and c) active disease at birth

Predictors of antibody positivity in colostrum	IgA (*n* = 158)	IgG (*n* = 160)	IgM (*n* = 76)
	**Odds Ratio (95% Confidence interval)**	**Odds Ratio (95% Confidence interval)**	**Odds Ratio (95% Confidence interval)**
Asymptomatic (*n* = 40, 42, 13)	Reference	Reference	Reference
Mild (*n* = 102, 104, 62)	1.27 (0.50, 3.04)	1.22 (0.15, 25.01)	0.80 (0.21, 3.97)
Severe (*n* = 16, 14, 1)	4.35 (0.72, 84.10)	3.15 (0.12; 83.62)	NA
Interval from disease to sample – colostrum (days)	1.00 (0.99, 1.00)	1.00 (0.98, 1.01)	1.00 (0.99, 1.00)
No active disease (*n* = 133, 136, 67)	Reference	Reference	Reference
Active disease (*n* = 25, 24, 9)	0.51 (0.20, 1.44)	1.43 (0.07, 10.25)	1.19 (0.16, 5.63)

*Ig* Immunoglobulin, Adjusted analyses were not possible due to small numbers. Numbers between parentheses are referring to the sample size of each stratum. For example, in the first line "(*n* = 40, 42, 13)" means that in the IgA model there were 40 asymptomatic women, in the IgG model 42 and in the IgM model 13

### Antibody evolution from colostrum to mature milk

In mature milk samples, IgG was positive in 2/62 (3.23%) (two women with active disease at birth that tested negative in colostrum); IgA was positive in 17/62 (27.42%) (32 women that tested positive in colostrum but negative in mature milk; *p*-value for the difference between IgA in mature milk vs. IgA in colostrum < 0.001, McNemar's chi-squared statistic = 29.032); and IgM was positive in 0/51 (6 of 51 were positive in colostrum).

## Discussion

### Main findings

The study has demonstrated that, firstly, all human breast milk tested for rRT-PCR SARS-CoV-2 are negative; secondly, antibodies against SARS-CoV-2 present in the colostrum do not seem to vary significantly in relation to the time when the infection has occurred during pregnancy or with regard to their presence in the maternal blood; and thirdly, IgA is the predominant immunoglobulin found in human breast milk and its concentrations are significantly lower in the mature milk compared with colostrum.

### Study strengths and limitations

To our knowledge, this is the largest series of colostrum samples from women with SARS-CoV-2 infection during pregnancy or at the time of birth (MEDLINE via Pubmed search (September 2023): ((Human breast milk[MeSH Terms]) AND ("COVID-19" [MeSH Terms])) AND ("antibodies" [MeSH Terms]), and where all three types of antibodies, as well as rRT-PCR-SARS-CoV-2, were tested. We also collected paired colostrum and mature milk samples and studied the serological status of the mother at the time of milk sampling, which allowed us to investigate the immunoglobulin association between the colostrum, mature milk, and maternal blood. Additionally, the protocol for collecting, handling, and storing samples was defined early and implemented in all centers [22]. Furthermore, we included 16 pregnant women with severe disease in the study, allowing us to investigate possible associations between the presence of immunoglobulins in colostrum and the severity of the disease.

The main limitations relate to the small sample size and the technical difficulties that further reduced the sample, which may have prevented us from recognizing other possible associations or significant findings. However, technical factors equally affect all samples, making it unlikely to be a source of bias. Besides, this study was conducted at the peak of the pandemic outbreak when vaccination was not a confounder, so the findings are still of great value. A second important limitation is that there is a wide range of gestational age at sampling, and the timing of colostrum and serum sample collection varied between days 0 to 4 postpartum, which may be responsible for physiological changes in immunoglobulin concentration. Nonetheless, we believe this also provides a better understanding of what happens during pregnancy and postpartum. Of note, there were fewer obese women and pregnancies ending in preterm birth than expected among infected COVID-19 pregnancies. However, this might be because most patients were recruited in non-tertiary referral centers, where the most severe cases were centralized.

#### Interpretation

It is well known that breastfeeding protects babies against gastrointestinal and respiratory infections [36–39]. IgA represents around 90% of all immunoglobulins in human milk, and its concentration is higher in the colostrum, decreasing during the first year of lactation [15]. Due to its low degradation and absorption rate in the infant's gastrointestinal system, IgA is the most important immunoglobulin in human milk since it protects the infant against infections at the mucosa level [16, 40]. Recently, it has been demonstrated that specific IgG, IgM, and IgA anti-SARS-CoV-2 antibodies in breast milk neutralize the virus in vitro [11, 41–43]. Therefore, anti-SARS-CoV-2 IgA in human breast milk could also protect the infant against the SARS-CoV-2 infection locally in their gastrointestinal mucosa, similar to what happens with other viral infections [44, 45].

In our study, most colostrum samples tested positive for IgA, irrespective of the time of SARS-CoV-2 infection. A significant reduction in IgA positivity was found when evaluating longitudinal changes in the colostrum and the mature milk. This is similar to what happens in other viral infections [15]. Importantly, IgA was present even in the colostrum of mothers with a negative serological status at childbirth, contrary to what happened with IgG, which was more likely to be detected when IgG in serum was also present. A possible explanation for this could be related to the fact that IgA is secreted from the maternal Gastrointestinal Antigen Linfoid Tissue (GALT) system and transported into the maternal mammary glands, where they are incorporated into the breast milk, while IgG is mostly filtered from the maternal plasma, albeit at a lower concentration [46]. When the infant nurses, they receive these antibodies along with essential nutrients from the maternal milk, providing passive immunity and protection against infections until their immune system matures [47, 48]. This system is responsible for secreting antibodies against common infections prevalent in maternal living area and, therefore, represent maternal memory [49]. This system also secretes IgM but at much lower concentrations.

In this study, 29 samples from women with active disease at childbirth were tested by rRT-PCR-SARS-CoV-2, and all were negative. Evidence suggesting the presence of SARS-CoV-2 in breast milk is conflicting [2–5, 8–10, 20], and it is possible that cross-contamination was responsible for the positive results [11]. Goad et al. investigated the presence of cell-specific expression of angiotensin-converting enzyme 2 (ACE2), proteases TMPRSS2, and cathepsins CTSB and CTSL in breast epithelium, and they did not find co-expression of ACE2/TMPRSS2 or ACE2/CTSB/L, which is essential for the entry of the virus into the cell. Therefore, they concluded that there was no risk of vertical transmission of SARS-CoV-2 in neonates through breastfeeding [50].

#### Clinical implications

This study confirms that SARS-CoV-2 is not detected in breast milk, even when active infection occurs at birth. Therefore, the possibility of vertical transmission while breastfeeding is extremely low. Furthermore, since antibodies are found in the colostrum irrespective of the time of infection, all women should be encouraged to breastfeed their infants, regardless of the time when the condition has occurred during the pregnancy, undertaking contact precautions when there is active disease. Nevertheless, since IgA concentrations drop significantly from the colostrum to mature milk, we could speculate that they might be even lower beyond six weeks postpartum, so public health measures should still be maintained to reduce the risk of the babies acquiring the infection.

## Conclusions

Our study has provided further evidence that breastfeeding is safe during maternal SARS-CoV-2 infection as the virus has not been detected in human breast milk, and protective antibodies have been found instead. However, larger studies with longer follow-ups are still needed.

### Supplementary Information

**Additional file 1:** **Table S1.** STROBE Statement-Checklist of items that should be included in reports of cohort studies.

**Additional file 2: Table S2.** Conformité Européene (CE) registration number of the reagents used for sample analyses.

## Data Availability

The data presented in this study are available on request from the corresponding author and conditioned to approval from the relevant Research Ethics Committees due to data protection regulations.

## Abbreviations

ACE2: Angiotensin-converting enzyme 2
BMI: Body mass index
CI: Confidence interval
ELISA: Enzyme-linked immunosorbent assay
GALT: Gastrointestinal antigen Linfoide tissue
IgA: Immunoglobulin A
IgG: Immunoglobulin G
IgM: Immunoglobulin M
IQR: Interquartile range
LMP: Last menstrual period
protS1: SARS-CoV-2 spike protein 1
RBD: Receptor binding domain
rRT-PCR: Real-time reverse-transcriptase-polymerase-chain-reaction
