# Supplementary material for: Anti-SARS-CoV-2-specific antibodies in human breast milk following SARS-CoV-2 infection during pregnancy: a prospective cohort study
Source: Int Breastfeed J. 2024 Jan 18;19:5. doi: 10.1186/s13006-023-00605-w (PMC10797875; doi:10.1186/s13006-023-00605-w)
Supplement: Supplementary file 1 — Additional file 1: Table S1. STROBE Statement-Checklist of items that should be included in reports of cohort studies. [file 13006_2023_605_MOESM1_ESM.docx]

**ADDITIONAL FILE**

**Table 1s. STROBE Statement**—Checklist of items that should be included in reports of ***cohort studies***

|  | **Item No** | **Recommendation** |
| --- | --- | --- |
| **Title and abstract** | 1 | (*a*) Indicate the study’s design with a commonly used term in the title or the abstract Done in the title |
|  |  | (*b*) Provide in the abstract an informative and balanced summary of what was done and what was found Done in the abstract |
| **Introduction** | | |
| Background/rationale | 2 | Explain the scientific background and rationale for the investigation being reported Done in the background |
| Objectives | 3 | State specific objectives, including any prespecified hypotheses Done in las paragraph of background |
| **Methods** | | |
| Study design | 4 | Present key elements of study design early in the paper Done in methods (Study population), first line |
| Setting | 5 | Describe the setting, locations, and relevant dates, including periods of recruitment, exposure, follow-up, and data collection Done, Second paragraph |
| Participants | 6 | (*a*) Give the eligibility criteria, and the sources and methods of selection of participants. Describe methods of follow-up Done, first paragraph |
|  |  | (*b*) For matched studies, give matching criteria and number of exposed and unexposed Not applicable |
| Variables | 7 | Clearly define all outcomes, exposures, predictors, potential confounders, and effect modifiers. Give diagnostic criteria, if applicable Done although |
| Data sources/ measurement | 8* | For each variable of interest, give sources of data and details of methods of assessment (measurement). Describe comparability of assessment methods if there is more than one group Done in methods |
| Bias | 9 | Describe any efforts to address potential sources of bias Done in methods |
| Study size | 10 | Explain how the study size was arrived at Done in methods |
| Quantitative variables | 11 | Explain how quantitative variables were handled in the analyses. If applicable, describe which groupings were chosen and why Done in methods (Statistical analysis) |
| Statistical methods | 12 | (*a*) Describe all statistical methods, including those used to control for confounding Done in methods (Statistical analysis) |
|  |  | (*b*) Describe any methods used to examine subgroups and interactions Done in methods (Statistical analysis) |
|  |  | (*c*) Explain how missing data were addressed Done in methods (Statistical analysis) |
|  |  | (*d*) If applicable, explain how loss to follow-up was addressed Done in methods (Statistical analysis) |
|  |  | (*e*) Describe any sensitivity analyses Done in methods (Statistical analysis) |
| **Results** | | |
| Participants | 13* | (a) Report numbers of individuals at each stage of study—eg numbers potentially eligible, examined for eligibility, confirmed eligible, included in the study, completing follow-up, and analysed Done in the first paragraph |
|  |  | (b) Give reasons for non-participation at each stage Done (Figure 1) |
|  |  | (c) Consider use of a flow diagram Done (Figure 1) |
| Descriptive data | 14* | (a) Give characteristics of study participants (eg demographic, clinical, social) and information on exposures and potential confounders Done Table1 |
|  |  | (b) Indicate number of participants with missing data for each variable of interest Done (Figure 1 and Table 1, 2 and 3) |
|  |  | (c) Summarise follow-up time (eg, average and total amount) Done in methods |
| Outcome data | 15* | Report numbers of outcome events or summary measures over time Done all through results |
| Main results | 16 | (*a*) Give unadjusted estimates and, if applicable, confounder-adjusted estimates and their precision (eg, 95% confidence interval). Make clear which confounders were adjusted for and why they were included Done in results (Table 4) |
|  |  | (*b*) Report category boundaries when continuous variables were categorized Done all through results |
|  |  | (*c*) If relevant, consider translating estimates of relative risk into absolute risk for a meaningful time period Not applicable |
| Other analyses | 17 | Report other analyses done—eg analyses of subgroups and interactions, and sensitivity analyses Done in methods (Statistical analysis) |
| **Discussion** | | |
| Key results | 18 | Summarise key results with reference to study objectives Done at main findings |
| Limitations | 19 | Discuss limitations of the study, taking into account sources of potential bias or imprecision. Discuss both direction and magnitude of any potential bias Done at study strengths and limitations |
| Interpretation | 20 | Give a cautious overall interpretation of results considering objectives, limitations, multiplicity of analyses, results from similar studies, and other relevant evidence Done at interpretation |
| Generalisability | 21 | Discuss the generalisability (external validity) of the study results Done at clinical implications |
| **Other information** | | |
| Funding | 22 | Give the source of funding and the role of the funders for the present study and, if applicable, for the original study on which the present article is based Done at funding |

*Give information separately for exposed and unexposed groups.

**Note:** An Explanation and Elaboration article discusses each checklist item and gives methodological background and published examples of transparent reporting. The STROBE checklist is best used in conjunction with this article (freely available on the Web sites of PLoS Medicine at http://www.plosmedicine.org/, Annals of Internal Medicine at http://www.annals.org/, and Epidemiology at http://www.epidem.com/). Information on the STROBE Initiative is available at http://www.strobe-statement.org.
