# Supplementary material for: Anti-SARS-CoV-2-specific antibodies in human breast milk following SARS-CoV-2 infection during pregnancy: a prospective cohort study
Source: Int Breastfeed J. 2024 Jan 18;19:5. doi: 10.1186/s13006-023-00605-w (PMC10797875; doi:10.1186/s13006-023-00605-w)
Supplement: Supplementary file 2 — Additional file 2: Table S2. Conformité Européene (CE) registration number of the reagents used for sample analyses. [file 13006_2023_605_MOESM2_ESM.docx]

**ADDITIONAL FILE**

**Table 2s.** Conformité Européene (CE) registration number of the reagents used for sample analyses.

|  | Order no. YG 0014-0101 |
| --- | --- |
| EUROIMMUN Analyzer 1 | Basic-UDl-DI 4049016YG0014G7 |
|  | Risk class A |
| ELISA Anti-SARS-CoV-2 (IGA) | 2606-9620 A |
| ELISA Anti-SARS-CoV-2 (IgG) | 2606-9601 G |
| ELISA Anti-SARS-CoV-2 QuantiVac (IgG) | 2606-9601-10 G |
| ELISA Anti-SARS-CoV-2 NCP (IgG) | 2606-9601-2 G |
| ELISA Anti-SARS-CoV-2 NCP (IgM) | 2606-9601-2 M |
| TaqPath™ COVID-19 CE-IVD RT-PCR Kit, 1000 Rxn | 64747 |
| Chemagic 360-D 2024-0010 | 60528 |
